# Mitochondrial dysfunction in cardiovascular disease: Towards exercise regulation of mitochondrial function

**DOI:** 10.3389/fphys.2023.1063556

**Published:** 2023-01-19

**Authors:** Kunzhe Li, Bingzhi Wan, Sujuan Li, Zhixin Chen, Hao Jia, Yinping Song, Jiamin Zhang, Wenyu Ju, Han Ma, Youhua Wang

**Affiliations:** ^1^ School of Physical Education, Institute of Sports and Exercise Biology, Shaanxi Normal University, Xi’an, China; ^2^ Physical Education Department, Xidian University, Xi’an, China

**Keywords:** cardiovascular diseases, mitochondrial dynamics, mitochondrial autophagy, mitochondrial energy metabolism, exercise

## Abstract

The morbidity and mortality of cardiovascular diseases are exceedingly high worldwide. Pathological heart remodeling, which is developed as a result of mitochondrial dysfunction, could ultimately drive heart failure. More recent research target exercise modulation of mitochondrial dysfunction to improve heart failure. Therefore, finding practical treatment goals and exercise programs to improve cardiovascular disease is instrumental. Better treatment options are available with the recent development of exercise and drug therapy. This paper summarizes pathological states of abnormal mitochondrial function and intervention strategies for exercise therapy.

## 1 Introduction

HF (heart failure, HF) remains a rapidly growing public health issue with an estimated prevalence of 40 million individuals globally (Baman and Ahmad 2020). An estimated 5.7 million additional United States. adults experienced heart failure between 2009 and 2012. The direct and indirect costs of Cardiovascular disease are increasing, making it a significant economic burden on society (Virani et al., 2020). According to the China Cardiovascular Disease Report 2021, cardiovascular disease prevalence and mortality in China are increasing, for about 330 million people now suffering from the disease. Cardiovascular diseases are the leading cause of death globally. The mortality rate of cardiovascular diseases is higher than that of tumors and other diseases. Four in every ten deaths is from heart disease a big public health problem plaguing nations around the world. The development of cardiovascular diseases has been systematically studied. Scholars suggested various models to explore the formation of cardiovascular diseases and the medical interventions to treat them.

**FIGURE 1:**
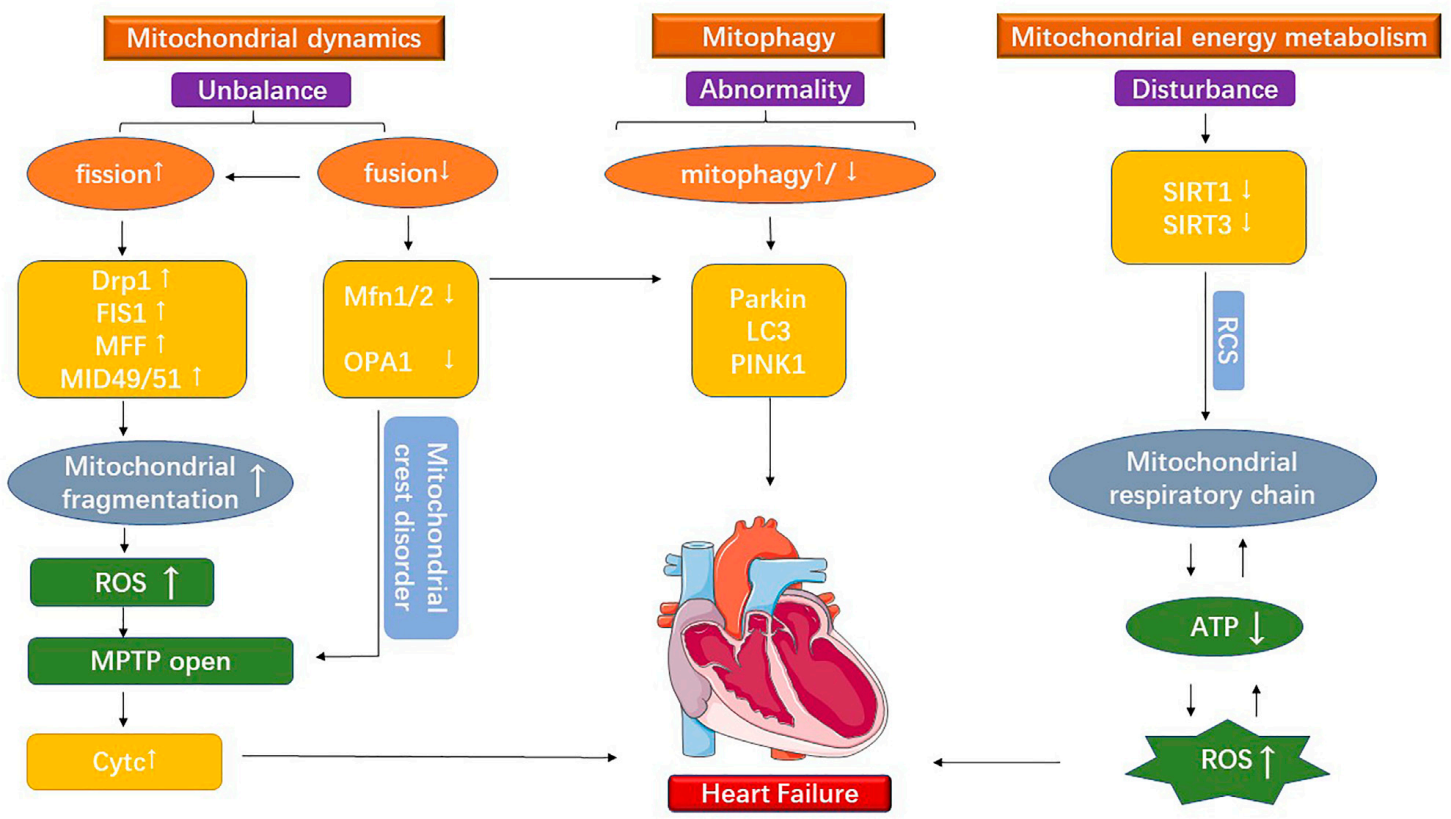
The figure shows the mitochondrial dysfunction under cardiovascular disease. It is divided into three main parts: mitochondrial division, mitochondrial fusion, and mitochondrial energy metabolism.

However, there is still a lack of effective therapeutic targets and rehabilitation therapies for treating cardiovascular disease. Moreover, the predisposing factors of cardiovascular diseases, such as hypertension, insulin resistance, diabetes, and obesity, are associated with imbalances in cardiac mitochondrial dynamics, mitophagy disorder, and mitochondrial metabolic dysfunction. Heart is one of the significant energy-consuming organs, containing about 30% of the mitochondria by volume of cardiac myocytes. Many findings showed that cardiac myocardial mitochondria imbalances are involved in different cardiovascular diseases and play a crucial role in their development (Huang et al., 2018; Wang et al., 2018; Maneechote et al., 2019). Michelakis et al. (2017) demonstrated that PDK (pyruvate dehydrogenase kinase, PDK) is a potential therapeutic target for improving patients’ blood flow dynamics by testing mitochondria-targeted human drug experiments. Zhou et al. (2020) contended that systemic inflammation in heart failure patients is closely related to functions such as mitochondrial respiration and energy metabolism. Besides, abnormal mitochondrial morphology and dysfunction are important markers of heart failure (Adaniya et al., 2019). Mitochondrial division interacts with mitochondrial fusion, while mitochondrial fusion, in turn, interacts with mitochondrial energy metabolism and mitophagy. Therefore, this paper discusses the molecular mechanisms of mitochondrial dysfunction in the development of the cardiovascular disease from the three aspects (Figure 1).

**FIGURE 2:**
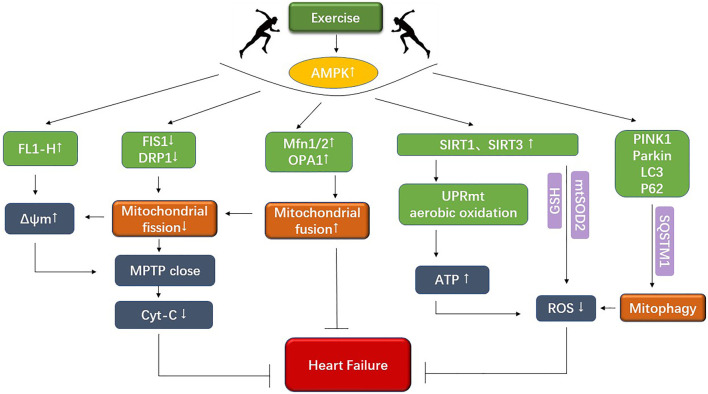
The figure shows that exercise improves mitochondrial dynamics as well as mitochondrial energy metabolism under cardiovascular disease.

Although there are many studies on pharmacotherapy for cardiovascular disease, many limitations and side effects are closely associated with the use of drugs. For example, defining the most appropriate outpatient dose of diuretic can be difficult. There is concern about vast individual variation, high therapy prices, and the impact on prognosis is unknown (Mullens et al., 2019). It is not widely used because of its limitations in practice and the need to constantly adjust the drug delivery schedule. Aerobic exercise costs less and has fewer side effects than most drugs. According to Fiuza regular exercise training directly benefits cardiovascular structure and function, reducing CVD risk, such as hypertension, dyslipidemia, and atherosclerosis of the blood vessels (Fiuza-Luces et al., 2018). Aerobic exercise interventions can regulate mitochondrial quality in pathological conditions, improve mitochondrial metabolic dysfunction, and mitigate the development of cardiovascular disease (Zhao et al., 2018a; Xiang et al., 2020). Another study showed that aerobic exercise could regulate ketone body and fatty acid metabolism through activating SIRT3-AMPK and SIRT1-PGC-1α pathways, inhibit the inflammatory response, and effectively improve the Warburg effect in cardiovascular diseases (Karbasforooshan and Karimi 2017; Xu et al., 2019). Among them, mitochondrial adaptation is one of the most critical causes of altered metabolic function (Xiang et al., 2020). At the same time, aerobic exercise can regulate mitochondrial kinetics, mitophagy, and mitochondrial energy metabolism by activating multiple factors and pathways, thereby improving the pathological state of cardiovascular disease. It is therefore argued that exploring the molecular mechanism of aerobic exercise to improve cardiovascular diseases can help find potential therapeutic targets for cardiovascular diseases and provide a theoretical basis for developing therapeutic strategies (Figure 2).

## 2 The biological mechanism of an exercise intervention to improve mitochondrial dynamics of cardiovascular disease

Steady-state mitochondrial dynamics implies a dynamic balance between the opposing processes of mitochondrial division and fusion, the loss of which can lead to abnormal heart physiological activity. Mitochondrial fission is a self-repair mechanism critical for regulating energy metabolism and maintaining mitochondrial function. Studies indicated that mitochondrial dynamics increase mitochondrial fission protein activity in myocardial hypertrophy and heart failure and decrease fusion protein activity (Maneechote et al., 2019). Moreover, mitochondrial fragmentation is found an essential trigger for increased ROS (Reactive oxygen species, ROS) and prolonged MPTP (Mitochondrial permeability transition pore, MPTP) opening under heart failure (Adaniya et al., 2019). Mitochondrial fragmentation-associated proteins such as Drp1 (Dynaml-related protein1, Drp1), Fis1 (Fission protein1, Fis1), Mff (Mitochondrial fission factor, Mff), and MiD49 and MiD51 (Mitochondrial dynamics proteins of 49 and 51 kDa, MiD49 and MiD51) were activated with the development of cardiovascular disease, suggesting increased mitochondrial fragmentation (Song et al., 2017; Adaniya et al., 2019; Konig et al., 2021). The prolonged opening of MPTP caused by mitochondrial fragmentation increases the permeability of the inner mitochondrial membrane, which causes many materials that are trapped initially to rapidly enter the membrane. Changes in mitochondrial osmotic pressure and the break down of outer mitochondrial membrane occur afterward (Xian et al., 2022). Recently, mitochondria-targeted nanoparticles (CsA@PLGA-PEG-SS31) have been used to target MPTP opening and inhibit cardiac function impairment (Zhang et al., 2019). Findings indicated that Drp1 has been implicated in the pathogenesis of cell death, accelerating the progressive development of dilated cardiomyopathy (Adaniya et al., 2019). To further demonstrate the vital role of mitochondrial breakage-mediated apoptosis in the development of cardiovascular disease. More studies argued that Drp1 is stimulated in cardiac myocytes injury caused by I/R (Ischemia-reperfusion injury, I/R) to induce increased mitochondrial division and aggravate mitochondrial fragmentation. At the same time, Fis1, Mff, and Mid49/51 engage in the mitochondrial division as receptors for Drp1 (Sharp 2015; Kalpage et al., 2019), causing the outer mitochondrial membrane to breaks down. The break-down outer mitochondrial membrane results in the release of cytochrome c from the inner and outer membrane gap, causing massive cell death and further worsening the pathological process of cardiovascular disease (Kalpage et al., 2019). However, reduced Drp1 also causes mitochondrial accumulation which induces cardiac hypertrophy and cardiac dysfunction (Shirakabe et al., 2016). In addition, the mitochondrial division-associated protein Drp1 is essential for autophagy during myocardial ischemia (Saito et al., 2019). These findings suggest that mitochondrial fission is of great importance for maintaining steady-state mitochondrial dynamics and supporting mitophagy. Further, it suggests an interactive relationship between mitophagy and mitochondrial dynamics. They work together to maintain the dynamic balance of the mitochondrial network.

Aerobic exercise is a promising intervention for improving mitochondrial fragmentation and mitochondrial fusion deficiencies in pathological states, restoring the balance of mitochondrial dynamics and ultimately slowing the progression of cardiovascular disease. It is suggested that aerobic exercise interventions play a significant role in the development of cardiovascular disease by regulating the homeostasis of mitochondrial dynamics. Previous studies by our research team have demonstrated that severe oxidative stress accompanies TAC-mediated heart failure (Ma et al., 2021). Among them, NLRP3, an important inflammatory factor, was significantly increased in TAC-induced heart failure, accompanied by the prolonged opening of MPTP mediated by upregulation of the mitochondrial fusion-related protein Drp1, which fully demonstrates that TAC-mediated upregulation of NLRP3 expression subsequently contributes to increased mitochondrial fragmentation. Moreover, aerobic exercise ameliorates cardiac impairment by modulating mitochondrial dynamics (Ma et al., 2021). It is demonstrated that aerobic exercise inhibited the phosphorylation of p-Drp1 at the Ser616 locus, increased Fis1 expression, reduced the occurrence of mitochondrial fragmentation, and restored the expression of mitochondrial fusion-associated proteins Mfn2 and OPA1 to normal levels in heart attacks, ultimately restoring mitochondrial kinetic homeostasis (Liu et al., 2019; Xue et al., 2019). Swimming exercise inhibits Drp1 expression and reduces mitochondrial breakage in cells, thereby improving blood pressure and vascular function (Li et al., 2022). ROS will lead to changes in mitochondrial fusion and mitotic protein activity. The imbalanced mitochondrial dynamics association will negatively affect the morphology and function of mitochondria through redox mediated signaling (Willems et al., 2015). The maintenance of mitochondrial integrity and homeostasis is extremely critical, which is achieved through continual fusion. The reduction of mitochondrial fusion induces cardiac myocyte hypertrophy and prolonged MPTP opening, ultimately leading to heart failure. Song et al. (2017) found that reduced activity of Mfn1/2 (Mitofusion 1/2, Mfn1/2), OPA1 (Optic atrophy 1, OPA1), OMA1 (Overlapping metalloproteinase associated protein 1, OMA1) and other related mitochondrial fusion proteins resulted in severe cardiac dysfunction (Guan et al., 2019). Scholars led by Papanicolaou et al. (2011)found that the absence of Mfn2 resulted in cardiac myocyte hypertrophy through an increased heart mass/body weight ratio in cardiac myocytes. In a mouse model of heart failure, over-hydrolysis of the mitochondrial inner membrane fusion protein OPA1 generate reduced mitochondrial fusion and indirectly increases mitochondrial fragmentation, resulting in mitochondrial dynamics imbalance and, ultimately, injury to cardiac function (Guan et al., 2019). OPA1 was overly hydrolyzed by activation of the OMA1 and MCU-OPA1 (Mitochondrial calcium uniporter, MCU) pathways under I/R, with a simultaneous increase in translocation of Drp1. Mitochondrial kinetic equilibrium is affected by the twin disruptions of mitochondrial division and fusion, and mitochondrial dysfunction develops over time to induce dilated cardiomyopathy and even heart failure (Dorn et al., 2015; Guan et al., 2019). Reduced mitochondrial fusion is complicated by cardiac myocyte hypertrophy, mitochondrial dysfunction, and apoptosis ender cardiovascular disease. Reduced mitochondrial fusion disrupts the overall balance of mitochondrial dynamics, thereby inducing prolonged MPTP opening leading to apoptosis and accelerating the progression of cardiovascular disease. It is becoming clear that the focus should be on mitochondrial dynamics-related regulators to maintain mitochondrial dynamics at a steady state to improve the biological mechanisms of cardiovascular disease.

Veeranki et al. (2016) found that exercise could improve mitochondrial membrane potential abnormalities by regulating the mitochondrial kinetics-related proteins Mfn2 and Drp1 to elevate JC-1 monomer FL1-H in cardiomyocytes. The correction of transmembrane potential could not only promote improved mitochondrial energy metabolism and prevent cytochrome c leakage in the cytoplasm, thus delaying apoptosis. It is suggested that exercise regulates mitochondrial dynamics, corrects transmembrane potential abnormalities, and reduces apoptosis by decreasing mitochondrial fragmentation. Redox processes are increasingly recognized as an integral part of the exercise-associated metabolism (Margaritelis et al., 2020). Exercise regulation of mitochondrial dynamics may affect mitochondrial redox metabolism, thus improving cardiac dysfunction (Kiyuna et al., 2018). Other studies have shown that AMPK can be activated by aerobic exercise, enhancing the expression of ADAMTS8, which encodes the expression of Mfn1/2, increases mitochondrial fusion, and restores the balance of mitochondrial dynamics, whilst reducing cardiomyocyte hypertrophy caused by insufficient fusion (Omura et al., 2019). Being physically active after a heart attack restored mitochondrial morphology for the elderly. It decreased Drp1 levels and mitochondrial fission on one hand, and increased OPA1 expression, mitochondrial fusion and mitochondrial dynamics on the other, according to research presented by Zhao et al. (2018a). Aerobic exercise can significantly reduce mitochondrial damage and ROS production. Meanwhile, it regulates the expression of Drp1, Mfn1/2, OPA1, and other proteins related to mitochondrial dynamics, which in turn regulate mitochondrial dynamics.

## 3 The biological mechanism of an exercise intervention to improve mitophagy and UPR^mt^ of cardiovascular disease

Mitophagy and the mitochondrial unfolded protein response (UPR^mt^) are the main protective mechanisms involved in repairing damaged mitochondrial. UPR^mt^ prevents abnormal protein accumulation within the mitochondria (Wang et al., 2021). Mitophagy is instrumental in the selective processing of damaged mitochondria (Zhang et al., 2017). As aging-damaged mitochondria are a significant source of ROS, the process of mitophagy is critical for maintaining the health of cells and the heart. More recent studies suggest that too much mitophagy or insufficient mitophagy could contribute to mitochondrial dysfunction and prolonged opening of MPTP, which causes heart failure (Wang et al., 2018; Maneechote et al., 2019; Fan et al., 2020). TAC-induced (Transverse aortic constriction) cardiac failure could reduce activity of LC3 (Light chain 3, LC3), PINK1 (PTEN-induced putative kinase 1, PINK1), and Parkin (Parkinson’s disease gene, Parkin). Thus, reduced mitophagy in pathological conditions represents a negative signal. Using the mitophagy inducer TB1 to increase mitophagy may slow the progression of heart failure (Wai et al., 2015; Wang et al., 2018). Induction of UPR^mt^ can alleviate cardiac dysfunction under myocardial hypertrophy (Xu et al., 2019). Cardiac dysfunction in MI rats was paralleled by increased protein levels of UPR^mt^ markers (Bozi et al., 2016). The downregulation of mitophagy and subsequent mitochondrial dysfunction is a crucial cause of accelerated cardiovascular disease pathology. During I/R injury, the fusion protein Mfn1/2 were inhibited, affecting Parkin-mediated ubiquitination and ultimately impairing inter-mitochondrial communication and cardiac function. Inhibition of p62 and LC3 binding and reduced mitophagy results in the prolonged opening of MPTP (Chen and Dorn 2013; Song et al., 2015; Cao et al., 2019; Maneechote et al., 2019). Reduced mitochondrial fusion exacerbates the inhibition of mitophagy in cardiovascular disease. Meanwhile, mediated mitophagy exacerbates oxidative stress, further reducing mitochondrial fusion factor activity. The two interact and form a vicious circle in pathological conditions, accelerating the development of cardiovascular disease.

However, excessive mitophagy could cause negative effects. According to Fan et al. (2020) in myocardial hypertrophy, oxidative damage in the body activates the PINK1/Parkin signaling pathway and induces mitophagy by increasing the expression of LC3-II. At the same time, mitochondrial damage, such as disturbed mitochondrial cristae morphology is also observed. Excessive mitophagy may lead to overwhelming mitochondrial damage and accelerate the progression from myocardial hypertrophy to myocardial fibrosis and heart failure. These findings suggest that excessive and insufficient mitophagy can lead to severe mitochondrial dysfunction and exacerbate the pathological process of cardiovascular disease. However, it is impossible to determine the stage of development of cardiovascular disease at this time by the growth or decline of mitochondrial autophagy. In turn, the process of accelerated cardiovascular disease by altered mitochondrial autophagy needs to be determined by a combination of simultaneous tests.

It has been demonstrated that aerobic exercise can regulate the level of mitophagy in pathological states, reduce the accumulation of autophagosomes, mitochondrial fragmentation and damaged mitochondria and alleviate mitochondrial dysfunction. By acting on factors associated with mitochondrial autophagy, these exercise-modifying effects modulate mitochondrial structure and function. In contrast, the heart is extremely rich in mitochondria, and there are multiple possible mechanisms by which exercise regulates mitochondrial function. Therefore, this study integrates several studies to discuss the potential pathways and targets as work to provide a basis for exercise interventions. To slow down cardiovascular pathophysiology process requires two parts: regulation of mitochondrial autophagy to clear damaged mitochondria by upregulating PINK1, a mitochondrial autophagy-associated protein in cardiomyocytes, which increases autophagosomes wrapped around mitochondria and can slow down the pathological process of cardiovascular disease (Zhao et al., 2018b). One of the possible mechanisms is to decrease AMPKα1 (AMP-activated protein kinase α1) by increasing AMPKα2 to promote Ser495 phosphorylation of PINK1, which in turn increases PARKIN to mitochondrial translocation and increases the rate of mitophagy. Also, upregulation of AMPKα2 could delay heart failure following TAC by activating myocardial mitophagy *via* the PINK1-PARKIN-SQSTM1 pathway (Wang et al., 2018). Exercise pretreatment may increase mitophagy levels in I/R by upregulating LC3-II and LC3-II/LC3-I to ensure regular cardiomyocyte activity and function (Gu et al., 2020; Shan et al., 2021). It has been previously clarified that prolonged MPTP opening causes apoptosis and cell necrosis, exacerbating cardiovascular disease. Previous studies found that aerobic exercise attenuated the increased susceptibility to MPTP opening, decreased the expression of LC3 -II, BAX, and mitophagy-related proteins PINK1, PARKIN, and p62, and increased the expression of Bcl-2(B-cell leukemia/lymphoma factor-2 gene). It is suggested that aerobic exercise may inhibit the development of cardiovascular disease by attenuating the susceptibility to MPTP opening and downregulating the expression of mitophagy-associated factors. Aerobic exercise training blunted MI-induced endoplasmic reticulum stress by reducing protein levels of UPR markers, and accumulation of both misfolded (Bozi et al., 2016). The above shows that exercise interventions not only play a therapeutic role in chronic heart failure, but also have a dual protective effect of preventing heart failure and reducing risk in the early stages. Accordingly, actively exploring the specific regulatory mechanisms and pathways involved will provide an experimental basis for clarifying aerobic exercise interventions in cardiovascular diseases and exploring potential therapeutic targets.

## 4 The biological mechanism of an exercise intervention to improve energy metabolism of cardiovascular disease

Mitochondria can oxidatively phosphorylate to synthesize ATP and use ROS to generate ATP. Mitochondrial dysfunction can cause reduced ATP production and oxidative stress caused by ROS accumulation (Picca et al., 2018). Mitochondria are wrapped by two membranes, where the inner membrane is folded and, these infoldings are referred to as cristae. The mitochondrial cristae represent the primary site of energy conversion and the residence of the respiratory chain complex and ATP synthase. Among them, OPA1 is centrally involved in the formation of the cristae (Kondadi et al., 2019). Basic research and clinical trials have shown that abnormalities in mitochondrial metabolism and mitochondrial dysfunction, such as the disappearance of the cristae and the destruction of the respiratory chain, can accelerate the progression of cardiovascular disease (Chen et al., 2018). Mitochondrial swelling and disruption of cristae morphology were observed in I/R and cardiac hypertrophy. In this case, the cristae morphology disorder leads to the release of cytochrome c, causing apoptosis of cardiac myocytes, which is an important feature in the progression of heart disease. Dysfunction of OPA1 causes the deregulation of cristae morphology which leads to the disruption of cristae morphology. Consequently, it affects the RCS (Respiratory chain complexes assembling into functional quaternary structures called supercomplexes, RCS) and the efficiency of mitochondrial respiration (Cogliati et al., 2013). It is revealed that pyridostigmine (pyridostigmine, PYR) facilitated mitochondrial fusion and improved the morphology and function of mitochondrial cristae by regulating the expression of OPA1, Mfn1/2, and the mitofilin/CHCHD3/Sam50 complex, which in turn reduced cardiac dysfunction (Xue et al., 2019). The clinically applied anticholinesterase drug PYR has shown improvements not only in neurological dysregulation of the autonomic nervous system, but also in mitochondrial abnormalities in cardiovascular disease. This suggests that mitochondrial medicine may have long worked through clinical drugs, but the exact molecular mechanisms underlying it are not yet clear. The mitochondrial fusion proteins OPA1 and Mfn1/2, which regulate the mitochondrial cristae, also contribute to mitochondrial energy metabolism. In addition, increased ROS production is observed in TAC-induced mouse hearts (Wang et al., 2018). Excess ROS interferes with the function of oxidized mitochondrial proteins. Reduced electron transport in the mitochondrial respiratory chain cause an increase in ROS production, resulting in oxidative stress. Growth of ROS production, combined with reduced electron transport in the respiratory create a wretched cycle (Zhao et al., 2021). It is suggested that the mitochondrial respiratory chain and dysfunctional mitochondrial metabolism are essential for the risk of oxidative stress in cardiovascular diseases.

In addition, some critical factors related to mitochondrial metabolic function have been identified in animal experiments. SIRT1 (Sirtuin1) is a potent anti-inflammatory factor of the, which regulates apoptosis as well as aging. SIRT3 (Sirtuin3) is emerging as a pivotal regulator of all signaling pathways related to cellular metabolisms in the human body, such as ROS production and detoxification, ketone body production, cell growth, and apoptosis (Watanabe et al., 2021). SIRT3 is distributed in several organs of the body, such as the heart and liver, which are highly metabolically active and are closely related to energy metabolism and oxidative stress. SIRT3 being a potential investigational factor could be an essential target for intervention in cardiovascular diseases. Studies have shown that SIRT3 KO mice disturbed fatty acid oxidation and reduced ATP levels during fasting (Yang et al., 2016). Also, decreased SIRT3 promotes ROS production, exacerbating the wretched cycle between oxidative stress and respiratory chain electron transport (Sun et al., 2018). SIRT1 functions as an anti-inflammatory factor and plays an active role in the disease, mitochondrial ROS-related factors in I/R injury are negatively correlated with the expression of SIRT1 (D'Onofrio et al., 2018; Prola et al., 2017). It indicates an interactive relationship between mitochondrial metabolic disorders and mitochondrial fusion. Other metabolism-related components involved in the pathological development of cardiovascular diseases are also demonstrated. However, the specific mechanisms are not fully understood and need to be explored in further studies.

Many studies have demonstrated that mitochondrial energy metabolism is closely connected to metabolic disorders in CVDs, such as pulmonary hypertension, cardiac hypertrophy, and heart failure (Kondadi et al., 2019). Among them, the War-burg effect is associated with increased dependence on anaerobic respiratory enzymes and the impairment of aerobic oxidative function. Aerobic exercise can regulate ketone bodies and promote lipolysis to increase free fatty acids and triglycerides in plasma, thus making more use of fatty acid metabolism. Consequently, the body is prone to be more dependent on aerobic oxidation for energy supply, improving the impaired energy metabolism in the disease (Xiang et al., 2020). Dajun Zhao et al. found that short-term exercise training after cardiac infarction increase mitochondrial SIRT3 expression and alleviate oxidative stress in aged hearts. The result is likely to be attributed to the improvement of mitochondrial dysfunction by regulating the ketone body and fatty acid metabolism through the SIRT3-AMPK pathway. Regulating mitophagy and activating UPR^mt^ in the heart can ultimately increases aerobic oxidative energy supply to increase ATP production, reduce the War-burg effect, and improve the pathological manifestations of myocardial hypertrophy (Xu et al., 2019). SIRT3 regulates metabolism and reduces oxidative stress damage under pathological conditions. Studies have shown that aerobic exercise can upregulate AMP/ATP ratio and SIRT3, activate AMPK phosphorylation and PGC-1α pathway, and reduces ROS and oxidative stress injury through enzymatic and non-enzymatic defense mechanisms (Bhatti et al., 2017). The AMPK-SIRT3 pathway is a potential therapeutic target for treating metabolic abnormalities and related cardiovascular diseases. The SIRT3-AMPK stimulate mitochondrial biogenesis can reduces myocardial injury (Xin and Lu 2020). Previously, mitochondrial cristae morphology was critical in mitochondrial energy metabolism and cell growth in cardiovascular diseases. Xue et al. (2019) found that exercise could improve the expression of AMPK, activate the LKB1/AMPK/ACC signaling pathway, and improve the morphology of mitochondrial crest, thus improving the stability of RCS and the efficiency of mitochondrial respiration. At the same time, mitochondrial cristae precipitate cytochrome c release and apoptosis (Bhatti et al., 2017).

Regarding the SIRT1 factor, exercise training has been found to enhance SIRT1 signaling to activate PGC-1α and enhance mitochondrial biogenesis, complementing metabolic signaling pathways and inhibiting inflammatory signaling pathways (Bhatti et al., 2017; Zhao et al., 2018a; Kornfeld et al., 2018). Melatonin significantly decreased Drp1 expression in the diabetic heart, decreasing mitochondrial division and thus delaying heart failure is also regulated through the SIRT1-PGC-1α pathway (Kornfeld et al., 2018; Wang et al., 2018; Jiao et al., 2021). Thus, the SIRT1-PGC-1α pathway may be a strategic target for cardiovascular disease treatment. It is sound and clear that aerobic exercise has played an essential role in energy metabolism disorders under cardiovascular diseases. In particular it is imperative to actively explore the regulatory mechanism of aerobic exercise on metabolism disorders under cardiovascular diseases to find effective therapeutic targets. Recent studies have found that exercise activates mitoAMPK, which regulates mitochondrial quality control and responds differently to energy stress in different networks. This suggests that targeting mitochondrial energy for disease treatment is particularly interesting (Drake et al., 2021).

## 5 Deficiencies in the application of exercise for cardiovascular disease in clinical practice

Exercise is widely recognized to improve cardiovascular disease, and the recent study of regulation of mitochondria by exercise has become a norm. Exercise can resist inflammation and improve myocardial metabolism. Mitochondria play an important role in the regulation of myocardial cell metabolism and inflammation. The regulatory targets of exercise and the specific clinical measures are still being researched and explored. There is still a lack of standardized and well-used exercise prescriptions. The New York Heart Association (NYHA) functional classification is commonly used clinically to classify patients with the cardiovascular disease into four classes. Exercise rehabilitation tools may not be available for all patients with cardiovascular disease, and each patient requires medical testing and exercise testing to reduce exercise risk, which is difficult and limited to existing medical conditions. Medical testing and exercise prescriptions for patients with cardiovascular disease require higher feasibility and better program planning. One study followed multiple patients with cardiovascular disease (NYHA classification I-III) and performed cardiopulmonary exercise testing (CPET), which included VE/VCO2 slope, peakVO2, and Predicted MVO2. The study confirmed that patients with NYHA levels I to III have some exercise capacity but that progression of cardiovascular disease is accompanied by a decrease in exercise capacity (Chelko et al., 2021). It is suggested that exercise intervention programs need to change according to the stage of development of cardiovascular disease. Other studies have shown that exercise-based cardiac rehabilitation improves health-related quality of life (HRQoL) and hospitalization rates in patients with heart failure. However, most trials were undertaken in patients with HF with reduced (<45%) ejection fraction (HFrEF), and women, older people, and those with preserved (≥45%) ejection fraction HF (HFpEF) were under-represented. Most of these trials were conducted in hospitals (Long et al., 2019). Similarly, one study found an improvement in HRQoL in heart failure patients who participated in the exercise, but there appeared to be no effect on mortality (Taylor et al., 2019). This shows that exercise rehabilitation for patients with cardiovascular disease is known to improve patient HRQoL and hospitalization rates but does not seem to significantly affect survival. The high intensity interval training (HIIT) and moderate intensity continuous training (MICT) have positive effects in heart failure patients. But HIIT has uncertainty and variation of actual training intensities (Gomes Neto et al., 2018). At the same time, most of the exercise rehabilitation interventions in these studies were conducted in specialized institutions such as hospitals, which are limited by the space and the professional staff. In addition, most exercise rehabilitation tools were individualized rehabilitation programs for patients’ exercise capacity after exercise testing, which indicates that exercise rehabilitation lacks uniform standards. These problems have hindered great difficulties to the application of exercise rehabilitation in cardiovascular diseases. Strengthening rehabilitation planning and implementation and specific exercise interventions are expected to be investigated further.

## 6 Conclusion

The pathogenesis of cardiovascular dysfunction is hugely complex. Mitochondria are membrane-bound cell organelles, playing an essential role in determining the development of cardiovascular disease. Essentially, mitochondria’s functional and kinetic homeostasis is necessary for normal cardiovascular function. Responses such as inflammation brought by mitochondrial structure and function damage can lead to the ultimate adverse outcome of cardiovascular dysfunction. In cases of disturbed mitochondrial dynamics, mitochondrial fusion and fission are in a state of imbalance. The absence of mitochondrial fusion and fission in dynamic equilibrium can lead directly to an exacerbation of the cardiovascular disease. The altered specific gravity of the mitochondrial energy supply pathway, the War-burg effect, is a direct killer of cardiovascular disease. Exercise can prevent the development of cardiovascular disease and improve the imbalance of mitochondrial dynamics and abnormal changes in mitochondrial structure in pathological states. Several studies showed that exercise rehabilitation can improve patients’ HRQoL and hospitalization rates. Meanwhile, exercise training is a type 1A intervention for treating cardiovascular disease risk. However, the implementation of exercise rehabilitation still lacks some theoretical basis and practical measures. Therefore, pharmacological and exercise interventions for cardiovascular disease are probably the most effective and practical treatment options. Exercise interventions to improve patient survival need to be further explored in clinical trials and molecular mechanisms.

## Data Availability

The original contributions presented in the study are included in the article/supplementary material, further inquiries can be directed to the corresponding author.
